# The plastid genome of *Pentadiplandra brazzeana* Baillon (Pentadiplandraceae)

**DOI:** 10.1080/23802359.2019.1688102

**Published:** 2019-11-12

**Authors:** Xu Zheng, Ting-Shuang Yi

**Affiliations:** a College of Life Sciences, Inner Mongolia Agricultural University, Hohhot, China;; b Germplasm Bank of Wild Species, Kunming Institute of Botany, Chinese Academy of Sciences, Kunming, China

**Keywords:** *Pentadiplandra brazzeana*, Pentadiplandraceae, Brassicales, plastome, phylogenomics, brazzein

## Abstract

*Pentadiplandra brazzeana* is the only species of Pentadiplandraceae. This species is shrubs or tuberous liana with a natural distribution in tropical West Africa. This study firstly determined its complete plastome. The plastome is totally 156,625 bp in length, which contains a pair of 26,751-bp-long inverted repeat regions (IRs), a large single copy region of 85,318 bp, and a small single copy region of 17,805 bp. A total of 112 unique genes were identified in this plastome, of which 78 are protein-coding genes, 30 are tRNA genes, and 4 are rRNA genes. Phylogenetic analysis based on 82 genes fully resolved relationships among sampled families of Brassicales, which are consistent with previous studies.

*Pentadiplandra brazzeana* is the only species of the family Pentadiplandraceae (Hall et al. 2004). It is an evergreen shrub or liana that is found in tropical West Africa, among northern Angola, eastern Nigeria and western Democratic Republic of Congo. It produces large red berries, sometimes mottled with gray. Its fruits contain two kind of high-potency thermostable sweet protein, called brazzein (Ming and Hellekant 1994) and pentadin (Vanderwel et al. 1989), which make the berry substantially sweeter than saccharose. Due to its sweetness, heat stability, high water solubility and minimum molecular weight, brazzein is the most superior protein sweetener presently known (Ming and Hellekant 1994). In this study, we determined the complete plastome of *P. brazzeana* based on the whole-genome Illumina sequencing dataset. We have deposited the annotated plastome of *P. brazzeana* in the GenBank with the accession number MN306574. Being the first complete plastome data for *P. brazzeana* as well as the family Pentadiplandraceae, the plastome sequence could be used for conservation and genetics studies of this endemic species, and the phylogenetic analysis of interfamilial relationships of the whole Brassicales.

The material of *P. brazzeana* used for this project has been collected from Kisangani, Democratic Republic of the Congo(0°31′0″N 25°12′0″E), and its specimen (Druk. 281/174) is deposited in the Royal Botanic Gardens, Kew. Total genomic DNA was obtained from the Royal Botanic Gardens, Kew. Paired-end sequencing was performed on Illumina HiSeq X TEN at Plant Germplasm and Genomics Center (Kunming Institute of Botany). The paired-end reads were filtered and assembled into a complete plastome using GetOrganelle v. v1.6.2a (Camacho et al. 2009; Bankevich et al. 2012; Langmead and Salzberg 2012; Jin et al. 2018), with final assembly graph checked using Bandage (Wick et al. 2015). The plastome was automatically annotated using PGA (Qu et al. 2019) and GeSeq (Tillich et al. 2017), then manually adjusted in Geneious 9.0.5 (Kearse et al. 2012). The plastome of *P. brazzeana* is 156,625 bp in length, containing a large single copy (LSC) region of 85,318 bp and a small single copy (SSC) region of 17,805 bp separated by a pair of 26,751-bp-long IR regions. The overall GC content was 36.3% and the plastome contains 112 unique genes (78 protein-coding genes, 30 tRNA genes, and 4 rRNA genes). Seven protein-coding, seven tRNA, and four rRNA genes are duplicated in the IR regions, the *ndhK* gene was pseudogene.

In order to verify the phylogenetic relationships of *P. brazzeana* in Brassicales, we constructed a maximum-likelihood (ML) tree of six important families of Brassicales that have plastome sequences, three representative species for Brassicaceae, and each one species for other five fimilies. The *Vitis rotundifolia* (Vitaceae) and *Bixa orellana* (Bixaceae) were used as outgroup (Figure 1). Phylogenetic analysis was performed on a data set including 82 gene sequences using RAxML version 8.2.11 with 1000 bootstrap replicates (Stamatakis 2014). Relationships among sampled families of Brassicales were fully resolved with strong supports at most nodes, which are consistent with previous studies (Edger et al. 2018).

**Figure 1.**
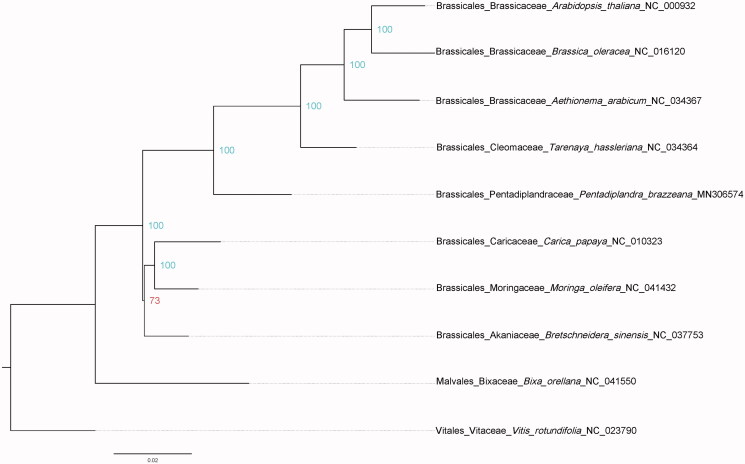
Maximum-likelihood (ML) tree inferred from the 82-gene (78 protein-coding and 4 rRNA genes) matrix. Numbers at the right of nodes are bootstrap support values. GenBank accession numbers: *Arabidopsis thaliana* (NC_000932), *Brassica oleracea* (NC_016120), *Aethionema arabicum* (NC_034367), *Tarenaya hassleriana* (NC_034364), *Pentadiplandra brazzeana* (MN306574), *Carica papaya* (NC_010323), *Moringa oleifera* (NC_041432), *Bretschneidera sinensi* (NC_037753), *Bixa orellana* (NC_041550), and *Vitis rotundifolia* (NC_023790).
